# Social, Family, and Educational Impacts on Anxiety and Cognitive Empathy Derived From the COVID-19: Study on Families With Children

**DOI:** 10.3389/fpsyg.2021.562800

**Published:** 2021-03-01

**Authors:** Alberto Quílez-Robres, Raquel Lozano-Blasco, Tatiana Íñiguez-Berrozpe, Alejandra Cortés-Pascual

**Affiliations:** ^1^Department of Educational Sciences, University of Zaragoza, Zaragoza, Spain; ^2^Department of Psychology and Sociology, University of Zaragoza, Zaragoza, Spain

**Keywords:** COVID-19, anxiety, cognitive empathy, families, education, social stressors

## Abstract

This research aims to monitor the current situation of confinement in Spanish society motivated by COVID-19 crisis. For this, a study of its socio-family, psychological and educational impact is conducted. The sample (*N* = 165 families, 89.1% nuclear families with children living in the same household and 20.5% with a relative in a risk group) comes from the Aragonese region (Spain). The instruments used are: Beck-II Depression Inventory (BDI-II); Baron-Cohen and Wheelwright’s Empathy Quotient (EQ) with its cognitive empathy subscale, as well as an ad-hoc questionnaire, reviewed by a panel of experts, to learn about socio-personal, family and housing conditions, use of technology, involvement in school tasks and household, and working condition. The multiple regression analysis results show that the anxiety derived from the current situation is explained in 23.1% (*p* < 0.001) by the variables: gender (t = -2.31, *p* = 0.022), level of Internet consumption (t = 2.139, *p* = 0.034), increase of family conflicts (t = 2.980, *p* = 0.003) and help with school tasks (t = 2.980, *p* = 0.040). On the other hand, cognitive empathy is explained in 24.6% (*p* < 0.001) by the variables: gender (t = -4.690, *p* < 0.001) and mother’s hours of teleworking (t = 2.101, *p* = 0.037). All this leads us to conclusions related to preventive systems of social, psychological, and educational aspects to better serve families. These conclusions can be also be transferred to the future with an inclusive care to family settings from those three parameters.

## Introduction

The current health emergency due to COVID-19 has led to numerous countries, and, above all, to Spain, to decree very drastic measures of house confinement (Gobierno de España, 2020). Life as it was known until now has been paralyzed. Only the work to produce essential services and products has been allowed and telework has prevailed over on-site work. All these measures have had an impact on people social, family, work, and educational life in the face of an unprecedented situation, which surely will have psychological and sociological consequences on the population, as well as changes in educational methods.

Recently, studies have been published that show how the COVID-19 pandemic is affecting the population health. The biopsychosocial model defends the need to attend not only to physiological symptoms (Guan et al., 2020; Holshue et al., 2020) but also to psychological and social elements such as: anxiety, depression, suicidal intentions, panic disorder, loneliness and psychotic signs (Moghanibashi-Mansourieh, 2020; Wang et al., 2020; World Health Organization, (WHO), 2020) caused both by the situation of isolation and by social consequences such as unemployment, inflation and closure of companies (Moghanibashi-Mansourieh, 2020). In his theoretical review Rajkumar (2020) shows the psychological consequences of this situation, highlighting how the incidence of anxiety and depression is increasing. Likewise, he argues the urgent need to implement specific interventions in vulnerable populations: children, adolescents and people with few resources. Although all age groups present at some extent psychological and social symptoms, age is a determining variable in the appearance and intensity of symptoms of psychological nature, and so is gender. In Iran, Moghanibashi-Mansourieh (2020) exposes how the level of anxiety in women is significantly higher than in men, also being higher in the group between 21 and 40 years old, predictably because this is the most economically affected group. Similarly, he indicates that the low level of education increases the anxiety rate, and, also, the continuous consumption of news related to the coronavirus. On the other hand, people who have relatives or acquaintances with COVID-19 are also severely affected by this type of psychopathology. Similarly, Wang et al. (2020) find that in China, women are the most affected in terms of psychosocial stress, anxiety and depression. It is necessary to contextualize anxiety and stress from the theory of family stress and coping theory (Patterson, 1988; Boss, 2001). In addition, these same authors, contemplate stress as a concept that is not always negative, and that obeys to the response of family adaptation in a communitarian and cultural context including factors or demands of normative and not thermal character, unresolved family tensions and small daily annoyances. Aligned with these studies, Taylor et al. (2008) recall how in the “flu epidemic” (pig flu) those people who lived in areas with a higher rate of cases suffered greater stress, being the youngest and less educated people the group with the highest risk of this disorder.

It is obvious that contextual elements play an essential role in anxiety control in situations of social isolation derived from natural, political or health emergencies, as is the case of COVID-19 crisis (Sugiura et al., 2020). In these situations, in addition to the main stressor, that is the crisis itself, authors such as Lock and Gordon (2012), add a series of secondary stressors derived from the family, social, economic, media or educational contexts that can aggravate these anxiety situations. These would be: economic stressors such as loss of income due to labor issues (Ehrlich et al., 2010; Picou and Hudson, 2010); stressors related to family or friends’ health during the emergency (Kun et al., 2010); stress related to exceptional education and schooling in these situations (Kilmer and Gil-Rivas, 2010); stress derived from exposure to catastrophic information provided by the media (Lau et al., 2010); stressors related to the increase in family conflicts (Irmansyah et al., 2010), understood as the disputes between parents, parents and children or among children that cohabitate (Emery, 1992), or greater burden on the distribution of household chores in some of the family members (McDermott, 2010); anxiety derived from the loss of social contact with friends and colleagues and, consequently, reduction of social capital (Wind et al., 2011); stress derived from loss of leisure and recreation (Irmansyah et al., 2010); and the anxiety related to changes in people’s opinions about the world they live in or about themselves (Lock and Gordon, 2012) towards a greater perception of the democratization of risk (Beck, 1998). To these secondary stressors, the publication by Howard et al. (2018) adds in the family context the fact of caring for the elderly, people with disabilities, having children under five years-old, having low incomes and belonging to groups with cultural and linguistically diverse backgrounds.

On the other hand, the empathy is an important construct in humanitarian crisis. This concept is defined as: “empathy allows us to interact effectively in the social world” (Baron-Cohen and Wheelwright, 2004, p.163). In this sense, social support among group members is related to higher levels of empathy in these crisis situations and, therefore, lower levels of anxiety both in who helps and who is helped, regardless of the personality associated factors (Maner and Gailliot, 2007; Siedlecki et al., 2014; Sugiura et al., 2020). This is because, in crisis situations, the human being is eminently social and cooperative (Glassman, 2000), so prosocial behaviors in emergency situations are common and have a positive effect on the psychological well-being of members of the affected community (Afifi et al., 2012; Welton-Mitchell et al., 2016). This aspect is especially important in the present situation of confinement, since families must not only care for their children, but also other family members who are ill (Porzio et al., 2020) or with psychological disorders (Chatterjee et al., 2020), who are more vulnerable to the Covid-19.

Regarding educational elements related to staying at home, it is necessary to refer to research related to home-schooling in which the fundamental difference with the previous situation is the free and responsible option of being at home exercising educational functions with the social and psychological repercussions that this entails. Petrovic and Rolstad (2017) allude to the philosophical part of the idea that from home education autonomy and citizen commitment can be addressed, aspects that Rousseau and Freire highlight (Molina, 2002). Together with these ideas, the bottom line is freedom and knowing how to combine them and create synergies between the individual and the social (Gofen, 2015). Although it is difficult to conduct scientific studies related to this type of teaching due to its own idiosyncrasy, we allude to questions that appear in the reviewed works. The first is that making home schooling compatible, regardless of whether guidelines are followed, ends up having better emotional and academic results (Neuman and Guterman, 2016, 2017). Authors like DeRish et al. (2020) agree on the relevance of external evaluations to students so that monitoring is better for families and for apprentices. At this point it is necessary to remember the value of the evaluation as a way of considering the real situation and improvement guidelines. Although other studies such as Cheng and Donnelly’s (2019) do not find significant differences in the emotional development and socialization of children schooled at home or in educational centers. However, it is decisive to keep in mind that anxiety in mothers who attend school at home can have a negative effect on attachment (Zajac et al., 2020).

Reference has been made to this situation of real isolation (intentional and obligatory), highlighting the repercussion on families at the social, work and educational levels, and this has been linked to anxiety and empathy as outstanding variables. Thus, anxiety is understood as a transitory emotion characterized by subjective feelings of tension and apprehension, which are consciously perceived, accompanied by hyperactivity of the autonomic nervous system that can vary in intensity and over time (Spielberger et al., 1982). On the other hand, empathy is defined as an ability to perceive, understand and share emotions and stands out for its role in maintaining relationships and prosocial behaviors (Haut et al., 2019). Therefore, its importance for family coexistence in the current confinement situation is highlighted.

The objective of the current research is: to study the impact at psychological, educational and socio-familial level derived from the COVID-19 pandemic in families. That is to say, to analyze the different contextual variables and their impact on the states of anxiety and cognitive empathy in confinement. The following hypotheses are expected to be tested: different socioeconomic variables directly affect the states of anxiety and cognitive empathy (Maner and Gailliot, 2007; Siedlecki et al., 2014; Sugiura et al., 2020). In addition, it is expected to find that specific variables such as work situation, distribution of household tasks, care of people at risk and help to children in educational tasks are the variables with greater predictive weight for empathy and anxiety (Ehrlich et al., 2010; Irmansyah et al., 2010; Kilmer and Gil-Rivas, 2010; Kun et al., 2010; Lau et al., 2010; Picou and Hudson, 2010). Finally, it is expected to prove a gender difference in terms of empathy and anxiety produced by the change in the variables previously mentioned (Moghanibashi-Mansourieh, 2020).

## Materials and Methods

### Sample

The sample is composed of 145 families from the Autonomous Community of Aragon (Spain), representing a total of 522 people, and with a family composition of 2 parents, or legal guardians with children, who respond the questionnaires. The average age of the adult participants is 42.52 (SD = 6.87) and 8.83 (SD = 6.32) for the children (Table 1). Of these, 32.4% had an only child, 63.9% two, and 4.7% three or more children. The selection was the result of a convenience and non-probabilistic snowball sampling on a national level, with a massive response from a single autonomous community (>90%), since the researchers belong to this region. The percentage of participation of families from other regions was very low (4.2%). Likewise, nuclear-type families exceeded 90% of the sample. It was decided to eliminate this data because it is not representative at a national level. The families excluded for not meeting the selection criteria represented 14.21% with a total of 87 individuals. The inclusion criteria were: (1) families belonging to the Community of Aragon, (2) families made up of 2 parents with children, (3) that they offer themselves (4) that the survey can be sent from the same address and in one go, and (5) that they send all the questionnaires duly completed. The questionnaires were completed during the first week of April 2020. The Spanish population was strictly confined for two weeks. It was measured at this time because we wanted to study the impact of this health measure, not its follow-up.

**TABLE 1:** Significate correlations of sociocultural variables and anxiety and cognitive empathy.

	**Anxiety**	**Cognitive Empathy**
Anxiety	1	0.183*
Cognitive Empathy	0.183*	1
Age	−0.257**	−0.182*
Gender	0.257**	0.399**
Number of children	0.004	0.166*
Children Ages	−0.167*	−0.186*
Father’s employment status	−0.091	−0.196*
Garden/Terrace	0.169*	−0.037
Increase of time watching TV	0.166*	−0.021
Increase of time using the Internet	0.302**	0.091
Mother’s hours teleworking	−0.002	0.236**
Time devoted to homework	0.229**	0.216**
Current help to children with school tasks	0.276**	0.251**
Past help to children with school tasks	0.163*	0.212*
Family conflicts	0.297**	−0.074
Household chores	−0.193*	0.024
Children’s use of technological resources	0.101	0.171*
Assistance to other at-risk persons	−0.026	0.184*
Mother’s help with school tasks	0.335**	0.238**
Father’s help with school tasks	0.164*	0.157

*p < 0.05, **p < 0.01.

### Research Process

The study received the approval of the Ethics Commission of the University of Zaragoza acting under the guidelines of the Ethics Committee of the Autonomous Community of Aragon. To do that, several phases were conducted that began in March 2020. In the first, information explaining the nature and objective of the project was launched massively on trusted social networks related to the educational field: schools, institutes and universities, as well as an invitation to participate to all those families that were interested. In a second phase, those who had expressed their willingness to participate were contacted by signing the informed consent, while ensuring confidentiality and anonymity that respected the ethical procedures of the Declaration of Helsinki (World Medical Association, 2001). The third phase consisted of sending the questionnaires (composed by *ad hoc* socio-educational questionnaire, Beck Anxiety Inventory (BAI) and cognitive empathy subscale of the EQ empathy quotient scale) that they had to fill in online and anonymously. The responses were recorded on a digital data storage base for later exporting.

### Instruments

*Demographic and socio-educational questionnaire ad hoc*, of own elaboration. The questions were related to the type of family structure, number of children, type of home, profession, employment situation during the crisis, family conflicts, household chores, school homework, etc., It consists of 25 items and was evaluated by a panel of experts, reaching an almost perfect level of agreement (k > 0.81) following the indications by Cohen (2013). Subsequently, to corroborate the conformity of the results, a post evaluation was established using an advisory council and following the criteria of the communicative methodology. With regard to the panel of experts, the level of agreement or concordance was calculated through the Kappa index, obtaining a value of 0.72 (”good” concordance force). A poor concordance is considered for values less than 0.20, weak of 0.21–0.40, moderate of 0.41–0.60, good of 0.61–0.80 and very good for values higher than 0.81 On the other hand, the internal reliability of the *ad hoc* survey was analyzed through Cronbach’s Alpha index reaching values of 0.62, with moderate to high reliability.

*Beck’s anxiety inventory (BAI)* in its Spanish adaptation by Sanz et al. (2005). It is designed for a collection of information in a self-report format that allows measuring the degree of anxiety and the aspects or symptoms less related to depression. Specifically, it measures anxiety derived from panic, panic and generalized anxiety disorders following the criteria established in the DSM-III-R. This questionnaire, dedicated to the measurement of cognitive and physiological anxiety consists of 21 items whose responses are evaluated from 0 to 3, being 0 “absolutely not,” and 3 “severely.” Reliability indices range from 0.41 to 0.58.

*Empaty Quotiente (EQ), Cognitive empathy scale* in its adaptation to the Spanish version (Redondo and Herrero-Fernández, 2018). The empathy quotient informs about the individual’s ease and willingness to capture and understand other people feelings and how they are affected by these feelings of others. In this case, the study focused on cognitive empathy, so only this subscale was used. It consisted of 7 items, valued between 0 and 3, being 0 “absolutely not,” and 3 “severely.” Cronbach’s alpha values for all subscales ranged from 0.67 to 0.80.

### Statistical Analysis

Statistical analysis was performed using the IBM SPSS Statistics for Windows software (version 25.0., IBM Cop., Armonk, NY, United States). First, a descriptive analysis of the socio-educational responses reported was performed. Its purpose was to describe the context of the current situation in qualitative terms. Subsequently, a comparison was obtained with the anxiety and cognitive empathy variables through analysis of variance (ANOVA). To establish the relationship between socio-educational factors and psychological factors of anxiety and cognitive empathy, a Pearson correlation analysis was carried out. To finish, a linear regression was performed to establish a predictive model of family and educational habits with the development of states of anxiety and empathy in the current situation of health crisis. All significant correlations were included in the regression model considering a value of *p* < 0.05 as statistically significant. On the other hand, linear regressions are carried out with steps forward. In this way, we can determine which element could be the most important.

## Results

### Socio-Educational Comparative Descriptive Analysis

The qualitative analysis of the socio-educational questionnaire yields the following results: Regarding the parents’ level of education, it is observed that mothers have university or postgraduate studies in a percentage of 65.7%, 30.8% have secondary studies, and 3.5% primary or basic studies. These data differ with respect to the fathers since 47% of them have university studies, 41.7% have secondary education studies and 11.3% elementary.

**FIGURE 1:**
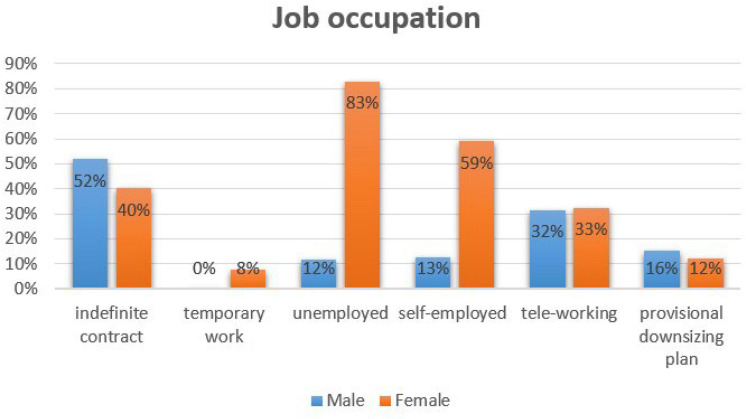
Progenitor employment situation.

Regarding their **job occupation** (Figure 1), the females recognize that 40.2% of them have an indefinite contract, and males the 51.8% of them. Temporary work is performed by 7.7% of women. In the self-employed modality, women are 5.9% and men 12.5%. On the other hand, 8.3% of the females are unemployed compared to the males, which represents a 1.2% and finally 2.4% of them are retired. Regarding the profession, 26% of women are civil servants, compared to 19.6% of men, and 9.5% are engaged in household chores work, that in the case of the males are not reported. These differences between genders are evident when conducting a business activity, where there are no reported data on women, but on the male gender it represents the 4.2%. These observations raise again the debate on the wage gap and equality and equity between genders. Training data is higher for women, but the unemployment rate, unstable employment, and occupation in household chores account for 25.5% of them.

About the **current employment situation** in the confinement situation, the results are similar between the genders with respect to telework (32.5% women; 31.5% men) and those affected by a provisional downsizing plan (12.4% women; 15.5% men). There is a difference in attendance at the workplace, where 17.2% of women do so compared to 28% of men.

On the other hand, the **economic situation** of families in the state of health alarm has undergone some modification. 59.6% of them say that the family economy has been affected during this exceptional situation, with the negative situation being of a serious nature in 27.8% of households.

Arrangement the average scores of the BAI scale, the results derived from the first 15 days of confinement showed a greater presence of cognitive-subjective stress and anxiety in the individual. In this sense, the mean anxiety (Mean:13.9, SD:9.95) during the period of confinement was significantly higher than that which would be expected to be found in the population, according to BAI criteria (Sanz et al., 2005). In other words, anxiety rates skyrocketed. This cognitive determinant is what made the type of empathy focus on the cognitive character and reject the emotional element. The average rate was (Mean: 13.12, SD: 3.87) a superior value in normal conditions (Redondo and Herrero-Fernández, 2018).

Regarding the **size of the house and the existence of a garden or terrace**, 76.3% reported living in a dwelling larger than 76 square meters (21.9% < 105; 34.9% between 76–90 and 19.5% between 91–105) and 14.7% of families occupied smaller houses. In addition, 37.3% of families did not have a large garden or terrace, which increases the possibility of feeling anxiety and seeing empathic development altered. For this reason, an analysis of variance (ANOVA) was performed, showing that there was a difference in the empathy developed at this time in relation to the size of the dwelling (*F* = 3.047; *p* < 0.05). Thus, people living in a space less than 60 square meters are significantly affected by the development of their empathy, obtaining an average score of 9 compared to 13.406 of the overall average score. On the other hand, no significant differences were found regarding the repercussion of this variable on the development of anxiety. Regarding the variable of existence of garden or terrace in the home, the opposite occurs since there are no significant differences with empathy, but they exist in anxiety, when increasing the feeling of confinement (*F* = 4.228; *p* < 0.05), being greater in those families that don’t have a terrace or garden (*M* = 15.463).

The results on the **use of leisure time** during the alarm situation caused by the pandemic, indicate that 63.8% of respondents admit to having increased television consumption, with the increase in the Internet consumption becoming even more drastic, as it was reported by the 95.3% of the sample (general family), and 29.6% of them assuring that this increase has occurred severely. Given these response data, a new ANOVA analysis was carried out on these variables and their link with anxiety and empathic-cognitive development, finding a strong impact on the increase in internet consumption with respect to anxiety (*F* = 4.779; *p* < 0.01). Families who claimed not to have increased their Internet use or to have done it very slightly reported mean anxiety scores of 7.285 compared to scores of 15.297 reported by those families who recognized a moderately high or severe increase of this consumption.

In the **educational field** and with respect to variables related to family hours devoted to children’s school tasks, 50.3% of the families affirm dedicating less than 4 h to these tasks. 17.8% of them affirm that they are spending between 4 and 6 h, compared to 7.7% who report a daily dedication of more than 6 h. In contrast, 24.3% of families do not spend any time monitoring their children’s homework. Differences in involvement between the maternal and paternal figure are observed. 41.7% of fathers claim not to help their children in the development of academic activities compared to 71% of mothers who do help their children. In addition, as a result of the current situation, family hours devoted to academic development of children has increased, going from 13% before the health crisis to 29.6% in the current situation, as they recognize helping in such tasks “always.” This greater involvement and dedication, to the detriment of the enjoyment of free time, can trigger the increase of anxiety states and empathic development. An analysis of variance finds significant differences that point to the possibility of a significant increase in both anxiety and emotional empathy (*F* = 3.565; *p* < 0.05 and *F* = 3.114; *p* < 0.05) related to this increase in parental involvement.

As for the variables related to **family coexistence** such as the distribution of household chores and the evolution or stagnation of family conflicts in the current situation, in 32.5% of the families the mother is still in charge of carrying out these tasks, compared to 6.5% of the father. However, 61% of families recognize that in the current situation of confinement, the distribution of tasks is more equitable, with 36.7% affirming that the tasks are carried out by both parents or legal guardians and 24.3% that the tasks are carried out by all the members of the family. Regarding the existence of family conflicts, 22.5% of the respondents report that they have increased and 27.2% of them that they have decreased. By linking these data with respect to the variables of anxiety and cognitive empathy, we found new differences in the ANOVA analysis, mainly with respect to anxiety (*F* = 6,276; *p* < 0.001), which increases in households where conflicts have intensified during the exceptional situation and decreases where tasks are equally distributed.

Finally, regarding the **existence of a person at risk** in the face of the health alarm caused by COVID-19 within the family nucleus, 20.7% of the respondents answered affirmatively, adding in 7.8% of the cases that these people also needed help. Another 27.8% of the sample had to attend someone at risk outside home. In this sense, an analysis of variance (ANOVA) determined the difference in terms of cognitive empathy with respect to this factor (*F* = 5.000; *p* < 0.005), turning out to be significant and establishing the increase in empathy in those who must assist people in risk at the present time. In contrast, no significant differences were found that affected the development of anxiety states.

### Correlational Statistical Analysis

To establish the relationship between socio-educational and psychological variables such as anxiety and cognitive empathy, a Pearson correlation analysis was performed (Table 1). A low positive significant correlation is found between these last two variables (0.183; *p* < 0.05). The results establish a significant negative correlation of anxiety and empathy with **age** (-0.257, *p* < 0.01; −0.182, *p* < 0.05), that is, the older the parents are, the lower the level of anxiety and empathy will be. **Gender** correlated significantly with anxiety (0.257; *p* < 0.01) and with empathy (0.399; *p* < 0.01), females have greater empathic capacity and greater anxiety. The **number of children** only correlates with empathy (0.166; *p* < 0.05), a higher number of children correlates with more empathy. In contrast, the **age of the children** correlates significantly and negatively with anxiety (-0.167; *p* < 0.05) and with empathy (-0.186; *p* < 0.05), the younger the children, the greater it is the parents’ level of anxiety and empathy. The **father’s occupation** only correlates significantly, low, and negative, with empathy. The higher father’s employment status is, the lower level of empathy towards the members of his family. On contrary, **mother’s hours of telework** presents a significant positive correlation with empathy (0.236; *p* < 0.01), indicating that the greater the number of hours teleworking, the greater her ability to empathize.

On the other hand, the time dedicated to **school tasks** by the children correlates significantly with anxiety (0.229; *p* < 0.01) and with empathy (0.216; *p* < 0.01). The same happens in the **assistance** provided by parents, at present time, to carry out these school tasks (0.276; *p* < 0.01; 0.251; *p* < 0.01) and the help provided before the crisis (0.163; *p* < 0.05;0.212; *p* < 0.05). The difference is that the **mother’s help** correlates significantly positively with anxiety (0.335; *p* < 0.01) and with empathy (0.238; *p* < 0.01), and **father’s help** correlates only with anxiety (0.164; *p* < 0.05). Parental involvement an increase in parents’ anxiety and, at the same time, empathy towards their children’s feelings. In turn, the time spent helping their children increases anxiety in the father and mother, but empathic development towards their children only occurs in the case of the mother. The variables of the existence of a **garden or terrace** in the home (0.169; *p* < 0.05), longer time watching **television** (0.166; *p* < 0.05) and increase in **internet** use (0.302; *p* < 0.01) only correlate with anxiety in a significantly positive way, being low for the first two, and moderate for the last one. On the other hand, **family conflicts** show a significant positive correlation with anxiety (0.297; *p* < 0.01). These variables pose a risk that negatively affects anxiety, altering family life. In contrast, the **children’s use of technological resources** correlates significantly with empathy and in a low positive way (0.171; *p* < 0.05), which implies an increase in empathy between them. Another variable that affects family life is the performance of **household chores**. This variable correlates significantly and negatively with anxiety (-0.193; *p* < 0.05), that is, with less collaboration from others, anxiety increases in the person in charge of them. Finally, **assistance to other at-risk persons outside family home** correlates significantly and in a low way with empathy (0.184; *p* < 0.05). Therefore, assisting people at risk outside home increases empathic capacity.

### Statistical Regression Analysis

Two regression analysis were conducted using the method of successive steps forward. The first was based on a predictive model of anxiety due to socio-educational causes in the context of current confinement (Table 2). The second elaborates a predictive model for the development of cognitive empathy in the same circumstances (Table 3).

**TABLE 2:** Regression model for predicting anxiety.

**Model**		**Standardized coefficients**	***t***	**Sig.**	**95% confidence interval for B**		
		**β**			**Lower limit**	**Upper limit**	**VIF**
1	Mother’s help with homework	0.335	4.25	0	2.501	6.849	1
2	Mother’s help with homework	0.303	3.955	0	2.112	6.334	1.015
	Increase of the Internet use	0.265	3.466	0.001	1.316	4.812	1.015
3	Mother’s help with homework	0.277	3.629	0	1.758	5.964	1.038
	Increase of the Internet use	0.242	3.179	0.002	1.056	4.532	1.034
	Gender	−0.176	−2.293	0.023	−7.006	−0.519	1.046
4	Mother’s help with homework	0.249	3.254	0.001	1.366	5.595	1.072
	Increase of the Internet use	0.252	3.341	0.001	1.189	4.637	1.038
	Gender	−0.186	−2.456	0.015	−7.215	−0.78	1.052
	Children’s average age	−0.151	−2.004	0.047	−0.445	−0.003	1.038

**TABLE 3:** Regression model for predicting cognitive anxiety.

**Model**		**Standardized coefficients**	***t***	**Sig.**	**95,0% confidence interval for B**		
		**β**			**Lower limit**	**Upper limit**	**VIF**
1	Gender	−0.399	−5.198	0	−4.814	−2.161	1
	Gender	−0.392	−5.269	0	−4.712	−2.141	1.001
2	Current help to children	24.	3.229	0.002	0.322	1.338	1.001
	Gender	−0.372	−5.029	0	−4.531	−1.974	1.016
	Current help to children	0.221	2.996	0.003	0.26	1.27	1.015
3	Mother’s teleworking hours	0,163	2.185	0.031	0.051	1.023	1.03
	Gender	−0.366	−5.022	0	−4.464	−1.942	1.017
	Current help to children	0.2	2.725	0.007	0.19	1.193	1.031
4	Mother’s teleworking hours	0.179	2.435	0.016	0.111	1.074	1.041
	Assistance to other at-risk persons out of home	−0.166	−2.27	0.025	−2.695	−0.186	1.025
	Gender	−0.377	−5.251	0	−4.536	−2.055	1.021
	Current help to children	0.159	2.154	0.033	0.045	1.056	1.085
5	Mother’s teleworking hours	0.178	2.452	0.015	0.114	1.059	1.041
	Assistance to other at-risk persons out of home	−0.185	−2.565	0.011	−2.848	−0.369	1.037
	Children’s average age	−0181	−2.467	0,015	−0.197	−0.022	1.064

In the anxiety prediction model, R^2^ values of 0.211 were obtained, which means an explanatory capacity on the variance of 21.1%. The variables that were significant for anxiety caused by the current situation were gender (β = 0.186; *p* < 0.05), children’s average age (β = 0.151; *p* < 0.05), mother’s help with homework (β = 0.249; *p* < 0.001) and the increase of the Internet use (β = 0.252; *p* < 0.001) (Table 2).

### Regression Model for Predicting Anxiety

For the predictive model of cognitive empathy, R^2^ values of0.274 were obtained, explaining the 27.4% of the variance. The significant variables for this model were gender (β = 0.377; *p* < 0.001), children’s average age (β = 0.181; *p* < 0.05), help in the current situation with homework (β = 0.159; *p* < 0.05), mother’s teleworking hours (β = 0.178; *p* < 0.05) and the fact of assisting other at-risk persons out of home (β = -0.185 y *p* < 0.05) (Table 3).

## Discussion

The main findings of this research are that: anxiety derived from the pandemic situation is explained by gender, the level of Internet consumption, the increase in family conflicts and help with homework. Cognitive empathy is explained by gender and mother’s teleworking hours.

The state of confinement has meant a new home structuring for families, becoming a place of work, upbringing, and care for both children (Zhang et al., 2020) and sick relatives (Chatterjee et al., 2020; Porzio et al., 2020). This study describes the current family situation where parents, in a 50%, have had to opt for teleworking or they are affected by a provisional downsizing plan and all children continue their studies at home through the use of ICT. It is also evident that, regarding the level of studies and type or modality of parents’ job, despite the fact that women have a higher level of education, they are also those who present the highest percentage in temporary jobs or unemployment. On the contrary, men are those who have indefinite jobs and conduct business activities. Regarding performing household chores and caring for people at risk, women still mainly do these tasks. These results are the consequence of a socio-cultural tradition that attributes stereotype and static roles based on gender (Blossfeld and Kiernan, 2019; McDermott et al., 2019). Regarding the variables that affect family life, the economic situation has been affected in more than the 50% of households, and television and internet consumption has increased as an alternative to outdoor leisure. On the other hand, it is shown that most of the respondents lived in houses of less than 76 square meters, but only a third of them did not have a garden or terrace. Regarding family conflicts, there is no unanimity, since, in the same proportion, they indicate that they have increased as well as decreased (between 20 and 25%), therefore it is inconsistent with studies that indicate that this type of situation generates stress that causes an increase in family conflicts (Irmansyah et al., 2010).

**Anxiety** is one of the main consequences of the COVID-19 pandemic, with the **females** and **young adults** being especially vulnerable (Moghanibashi-Mansourieh, 2020; Rajkumar, 2020; Wang et al., 2020). In this sense, older age means better crisis management (Kang, 2014), being data that are aligned with our results. To the above we must add that the lack of a garden or terrace that supposes a greater feeling of confinement, the increase in hours watching the television as an alternative to outdoor activities, dedicating more hours to carrying out school tasks and helping children with them (to a lesser extent by fathers), and an increase in family conflicts, lead to intensification of anxiety levels in confined persons. These data are in line with those provided by Lock and Gordon (2012) since the crisis situation is aggravated by effects derived from the family context and with those by Irmansyah et al. (2010) related to the loss of hours of leisure and outdoor recreation. On the other hand, the **predictive model** exposes that not only **gender** is an explanatory element, since there are other contextual and socio-family elements that can significantly affect stress and anxiety in this type of situation, as suggested by Sugiura et al. (2020). For example, the **children’s average age** (the younger they are, the higher parents’ anxiety levels), **mother’s help in school tasks** and increase in the **use of Internet** are factors that also influence the development of states of anxiety in the current situation. These elements are aligned with previous research, in which pointed out as stressors we find: having children under 5 years-old (Howard et al., 2018), exceptional education and schooling in these situations (Kilmer and Gil-Rivas, 2010), or the unequal distribution of household chores (McDermott, 2010). Regarding the role of the Internet in this process, it is necessary to reference how its remarkable growth has been paired with alarming figures of addiction worldwide, and its consequences in the increase of depression and anxiety (Cheng and Li, 2014; Al Mamun and Griffiths, 2019; Lozano-Blasco and Cortés-Pascual, 2020). The Internet is the key to a new environment where a multitude of activities are carried out, from shopping to establishing affective relationships (Kiraly et al., 2015) and in a confined environment, networks offer the maintenance of lost daily life: work, consumer goods and services, or attending to the education of children. Although, it should be borne in mind that norm-typical human behavior can be altered by its continuous interaction in the cloud (King et al., 2013; He et al., 2017).

On the other hand, the highest levels of **empathy**, in crisis situations, are related to the greater support among the members of the family unit, which is generated especially in those who carry out a greater proportion of help, work or assistance (Siedlecki et al., 2014). In these types of situations, a positive effect arises in those with the highest cooperative commitment (Glassman, 2000). In this sense, the results obtained in this research coincide with these previous studies since, as indicated above, being mother, having younger children, as well as their number, mother’s teleworking hours, time helping with school tasks (especially for the mothers), having technological resources and assistance to people at risk outside home, are positively associated with empathy. The **predictive model** indicates that cognitive empathy is modulated by gender (greater empathy in women), the average age of the children (the younger the children, the greater the empathy), current help with school tasks, assistance to people at risk outside home and mothers’ hours of teleworking, variables that increase these levels of empathy. Empathy within parental relationships is explained by the strength of the relationship between them and by the generation of response to affective states, being in full agreement with research such as the one by Eisenberg and Miller (1987) and Verhofstadt et al. (2008). On the other hand, it has sometimes been found that the level of education predicts the level of empathy (Haut et al., 2019) and in the present study it has already been pointed out that the sample formed by females had higher academic levels than males.

In relation to the first two variables, gender and age of the children, there would be a correspondence with the levels of anxiety, since, as specified, the fact of being a woman and having younger children increases anxiety (Howard et al., 2018; Moghanibashi-Mansourieh, 2020; Rajkumar, 2020; Wang et al., 2020), but empathy also increases with these variables, explaining the correlation established between both variables of anxiety and empathy. Regarding the variables related to help or collaboration with others, assistance to other people-at-risk outside home and the help to children with school tasks are key factors in modulating cognitive empathy, so that it is increased in these situations, but almost exclusively in females. In this sense, although authors such as Maner and Gailliot (2007), Porzio et al. (2020), Siedlecki et al. (2014), and Sugiura et al. (2020) point out that assisting family members can be a major stressor in emergency situations (also in the case of our study, where the mother’s school help was also an anxiety factor), in general, publications on this matter have been focused on how the social support network (family, friends, community members) is an element of great relevance when managing stress situations derived from different types of crises. These two examples of prosocial behaviors, common in emergency situations (Afifi et al., 2012; Welton-Mitchell et al., 2016), are a factor promoting empathy and cooperative spirit in the face of adversity (Zakour and Gillespie, 2012; Kim and Zakour, 2017). In other words, the human being has the ability to increase his cognitive empathy in the moments of greatest need in crisis situations (Glassman, 2000), seeking the psychological well-being of the group (Afifi et al., 2012; Welton-Mitchell et al., 2016) having a positive effect on both the person helping and the person being helped (Maner and Gailliot, 2007; Siedlecki et al., 2014, Sugiura et al., 2020). This last element is verified in our study in the increase of empathy when helping children in school tasks. Lastly, the mother’s hours of teleworking seem to be an element that promotes empathy, and can be related to the fact of not having lost a job and having a greater ease of reconciling work and personal life in an emergency situation, being aligned with studies such as those by Ehrlich et al. (2010), Howard et al. (2018), and Picou and Hudson (2010).

Regarding the limitations of the study, the urgency of the situation, the sampling method, and the reception of a majority response from only one region in Spain can cause the sample to be slightly biased. However, the results present optimal indicators and seem to be aligned with the literature (Glassman, 2000; Maner and Gailliot, 2007; Afifi et al., 2012; Siedlecki et al., 2014; Welton-Mitchell et al., 2016; Sugiura et al., 2020). Regarding prospective, a repetition of the study once the period of confinement has ended and the population will adapt to the new normality, would serve to verify whether this crisis has changed the socio-family dynamics and the levels of empathy and anxiety of the population.

We consider what response we give from education, science, and society to attend these moments of emergency and that are linked to normative or cultural elements. It would have to be transversal, regardless of the moment in which one lives, empathy towards the complex situations of other people, the search for emotional balance, compassion and actions linked to justice and social well-being. In and after this crisis, we can take many positive aspects to implement in families and in society, such as knowing how to work as a family team, respecting emotional singularities and looking for the contagion effect to the rest of social areas. An example of this is the librosqueunen.org project in which a bond is created with the family in a situation of educational disconnection due to the digital divide. It has been proven that we are able to increase our empathy and regulate our anxiety, although we need more formal and non-formal education in this line, taking into account all the factors that are included in a family, and that are not only the family members or the “school inside the house” elements, but also the technological, media and social networking sites.

## Conclusion

With this research we approach the current situation of confinement in Spanish society motivated by COVID-19 crisis. For this, a study of the social, psychological, and educational impact on families is conducted. Undoubtedly, the results highlight the inequalities that still exist within the family sphere. Derived from our data, we could hypothesize that the mother figure continues to bear the burden of work at home and she is the sustenance that maintains family relationships and harmonious coexistence. The father figure continues to establish his work relationships as a priority, occupying a secondary place in the relationship with children and adults. Anxiety increases in strange situations in this context and empathy, especially by females, dampens the stress caused. Although authors such as Howard et al. (2018) and Sugiura et al. (2020) list a greater variety of stressors related to the personal, family, social and work environment than those identified in this study, the uniqueness of the global emergency situation caused by the COVID-19 crisis, justifies that the assimilation of this situation to other previous emergencies is not possible. Although many of the studies collected in this research analyze exceptional situations (health emergencies, natural disasters, socio-political conflicts), the characteristics and global nature of this crisis places us in a new scenario, in which the truths assumed up to now are questioned. As Beck (1998) stated in the current Risk Society these new threats, derived from human action, shake the central components of society, motivating a series of debates and reformulations that science and society must jointly explore.

## Data Availability Statement

The raw data supporting the conclusions of this article will be made available by the authors, without undue reservation.

## Ethics Statement

The studies involving human participants were reviewed and approved by CEICA (Comité Ético de Investigación Clínica en Aragón) del Gobierno de Aragón and Universidad de Zaragoza (ACUERDO de 2 de abril de 2020, del Gerente de la Universidad de Zaragoza, por la que se aprueba el Tratamiento de datos personales “Monitorización del Covid-19: Impacto social, psicológico y educativo”). The patients/participants provided their written informed consent to participate in this study.

## Author Contributions

AQ-R coordinated the design and research process. AQ-R and RL-B designed the psychological research. TÍ-B designed the sociological questionnaire. AC-P the educational one. AQ-R and RL-B carried out the statistical analysis. RL-B, AC-P, and TÍ-B carried out the theoretical framework, discussion and conclusion. All authors contributed to the article and approved the submitted version.

## Conflict of Interest

The authors declare that the research was conducted in the absence of any commercial or financial relationships that could be construed as a potential conflict of interest.
